# Chemical Burns Caused by Topical Silver Nitrate in Umbilical Granuloma Treatment: A Case Report

**DOI:** 10.7759/cureus.76041

**Published:** 2024-12-19

**Authors:** Muhammad M Saleem, Mishal Pervaiz, Ismail Mazhar, Uswah Shoaib, Muhammad Ibrahim Tahir, Haseeb Ahmad, Sehar Khauteja Khan, Khawaja Haider Sami

**Affiliations:** 1 General and Pediatric Surgery, Combined Military Hospital (CMH), Lahore, PAK; 2 Anesthesiology, Combined Military Hospital (CMH), Lahore, PAK; 3 Pediatric Surgery, Combined Military Hospital (CMH) Lahore Medical College, Institute of Dentistry, Lahore, PAK; 4 Internal Medicine, Combined Military Hospital (CMH) Lahore Medical College, Institute of Dentistry, Lahore, PAK; 5 Pediatric Surgery, Lahore Medical and Dental College, Lahore, PAK

**Keywords:** benign condition, granulation tissue, infant umbilical granuloma burn injury, neonates, umbilical stump

## Abstract

An umbilical granuloma (UG) is one of the most common umbilical anomalies seen in infants, mostly due to delayed cord separation. It is usually treated with silver nitrate; however, topical application of silver nitrate can cause chemical burns, creating concern among parents. We present a similar case in a two-month-old baby boy with a giant UG, which was treated with topical silver nitrate application, producing significant chemical burns around the umbilicus and on the anterior abdominal wall. The patient was treated conservatively with a good outcome.

## Introduction

Umbilical granuloma (UG) is a common benign condition in neonates presenting as a moist, fleshy, pale-red granulation tissue mass at the umbilical base after cord detachment [1]. Typically soft, vascular, and 3-10 mm in size, UG often appears with seropurulent discharge due to irritation or secondary infection. It results from incomplete epithelialization of the umbilical stump [2]. Global incidence ranges from 1% to 12.8%, with annual rates being 3.8% to 7.3% among neonates [3]. Factors such as male sex, longer gestation, and meconium-stained amniotic fluid are linked to UG development, though the precise etiology is unclear. Environmental factors like heat and humidity may further promote UG formation following cord detachment. Management options include surgical and non-surgical approaches, with topical steroid application and silver nitrate cauterization being widely used. We present a case of chemical burns following silver nitrate application to a large UG, which was managed conservatively without significant morbidity.

## Case presentation

**Figure 1:**
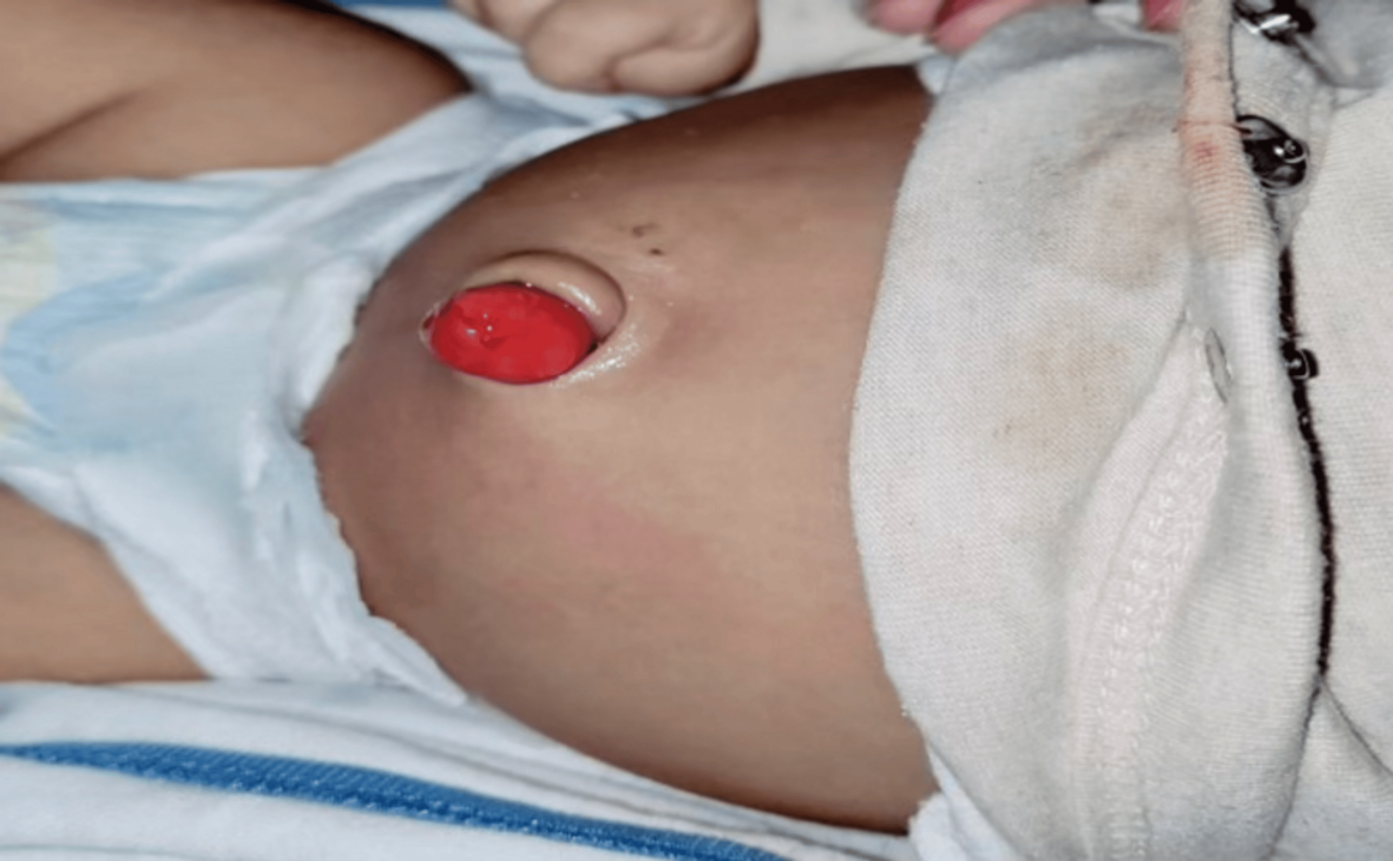
Large polypoid growth at umbilicus on presentation

A two-month-old male infant, weighing 3.8 kg and born at term via spontaneous vaginal delivery with an unremarkable antenatal history, presented to the outpatient department with swelling at the umbilicus. The mother first observed the mass following the separation of the umbilical cord, which occurred on the sixth day of neonatal life. Since then, the swelling has progressively enlarged, with no associated fecal or urinary discharge. On general examination, the child appeared healthy and thriving. Local examination revealed a 4 cm polypoid, pinkish mass at the umbilical region, accompanied by mild mucoid discharge, while the surrounding skin appeared normal (Figure 1).

**Figure 2:**
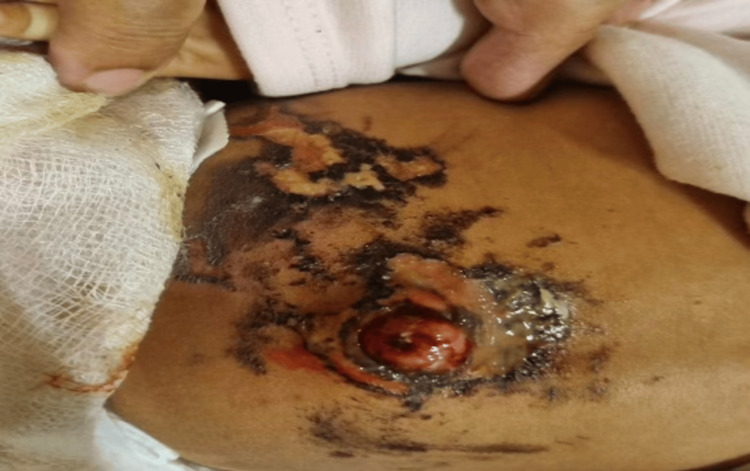
Superficial chemical burns with blackish discoloration

A clinical diagnosis of UG was made, and an ultrasound of the abdomen and pelvis was done to rule out any communication with the gut and urinary bladder. It was initially treated with topical steroids for two weeks. On follow-up, parents reported that the granuloma size had further increased. It was then decided to proceed with silver nitrate cautery as an outdoor procedure using 20% concentration. The surrounding area was covered with Vaseline to prevent direct contact of silver nitrate with the surrounding skin. The baby was kept for one hour post-application and was sent home with instructions to follow up after five days. The parents presented again on the same evening with multiple blackish discoloration areas over the swelling, around the periumbilical area, and around the anterior abdominal wall, along with exudative ooze from the granuloma (Figure 2).

**Figure 3:**
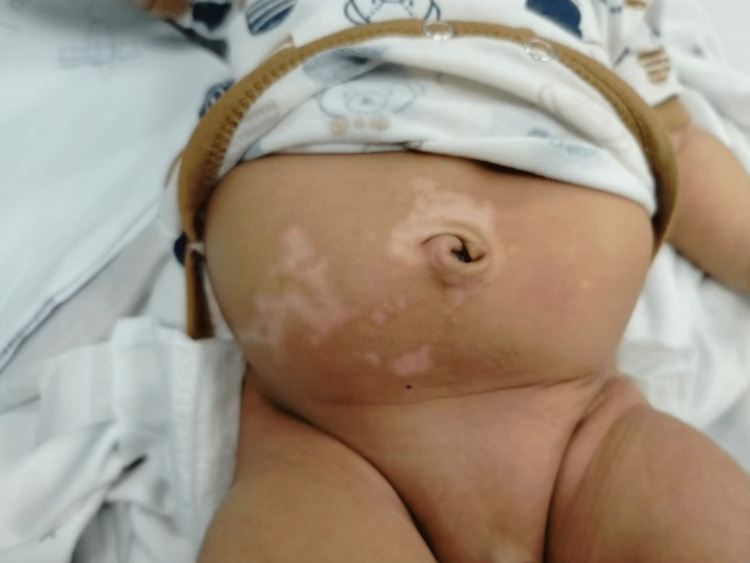
Healed burn wounds on follow-up

The patient was admitted after his parents' counseling and reassurance. Copious saline wash with curettage of the burn area was done, followed by sterile paraffin gauze dressings in the minor room. The base of the granuloma was tied with polyglactin 4/0 suture after giving local anesthesia. An excised specimen was sent for histopathological examination. Reassessment revealed approximately 2% superficial burns involving mainly the right side of the umbilical area. Oral analgesics and antibiotics were advised for pain and to prevent secondary infection. Burn wounds improved markedly following three sessions of sterile paraffin gauze dressings on alternate days, and the baby was discharged. A three-month follow-up showed a completely healed wound with no active complaints (Figure 3).

## Discussion

Silver nitrate cauterization is one of the most commonly used treatments for UG; although effective, it requires careful application and parental counseling regarding chemical burns and hyperpigmentation, which typically resolve within 12 weeks depending on the concentration used and are rarely reported in the literature [4]. Kesaree et al. demonstrated the effectiveness of silver nitrate topical use for UG treatment in 1983, and since then, it has been used in all parts of the world for the said purpose [5]. As a cauterizing agent, silver nitrate releases silver ions that bind to tissue to form an eschar, occluding vessels and thus producing shrinkage in UG size. However, it can damage adjacent tissue on contact and carries the potential risk of chemical burns to areas like the periumbilical region or abdominal wall, as was in our case [6]. Applying petroleum jelly, vaseline, or liquid paraffin to safeguard the surrounding skin during treatment may not help in preventing this potential complication, as the exudate produced by the UG can be extensive and can involve skin beyond the protective layer applied. Although silver nitrate cautery offers the advantages of a high healing rate for UG and low cost, its potential to produce chemical burns is a concern among medical professionals and should be applied by trained medical professionals in a hospital setting. Parents should be informed beforehand about this risk in detail.

There is no consensus on the first-line modality for UG treatment. Topical steroids, such as clobetasol and betamethasone, can serve as alternatives to silver nitrate, offering comparable healing rates. [7]. We opted for this management as first-line therapy in our case and used silver nitrate after a poor response to steroids. Surgical excision, although suitable for larger or refractory granulomas, is impractical for routine use due to the need for general anesthesia [8]. The extent and severity of burns can be related to UG size and the concentration of silver nitrate used. Say reported a case of chemical burns caused by topical silver nitrate at 75% concentration, which led to more severe and extensive burns in an infant [9]. Although we used a 20% concentration, the larger burn area may be attributed to the significantly larger UG, which resulted in excessive reactionary fluid in response to the applied silver nitrate. Another case was reported recently by Ho and Huang involving a 19-day-old baby girl, where 20% silver nitrate used for UG treatment caused superficial periumbilical chemical burns. Conservative management yielded promising results and no residual deformity, similar to our case [10]. Majjiga et al. reported a similar incident of chemical burns in a neonate treated for UG by a general practitioner, who later presented with hyperpigmentation and periumbilical burns that were managed conservatively [11]. Additionally, a case of skin corrosion and discoloration following silver nitrate use was reported in 2016 by Winther et al. [12]. Therefore, silver nitrate should be used cautiously, with prior parental counseling and education regarding the rare and superficial chemical burns that can lead to significant anxiety among parents and unnecessary hospital admissions requiring repeated dressings and prolonged treatment duration.

## Conclusions

Despite limited evidence on the optimal treatment for umbilical granulomas, a systematic medical history and physical examination allow for individualized treatment decisions, taking into account the family's and healthcare center's resources. Silver nitrate remains the first-line treatment in clinical settings; however, its astringent and caustic effects necessitate cautious use under supervision. Even with all precautions, the occurrence of chemical burns cannot be entirely predicted, although silver nitrate generally provides good long-term outcomes. Alternative options, such as salt application, should be considered, with surgical interventions like ligation or excision reserved for persistent cases. Additionally, poor hygiene and delayed medical care may lead to severe infections, such as omphalitis or sepsis.
